# 

**Published:** 1883-03

**Authors:** 


### CONTENTS.

### *MISCELLANEOUS.*

| | PAGE |
|---|---|
| A New Homœopathy. Editorial, | 73 |
| Correspondence of the New York Medical Times. P. P. Wells, M. D., | 74 |
| Drs. Sayre and Jacobi on Homœopathy and Homœopathists. Extract, | 81 |
| The Central N. Y. Homœopathic Medical Society, | 84 |
| International Hahnemannian Association, | 88 |
| Some Characteristic Symptoms, | 89 |
| Tait's Operation. Extract, | 91 |
| Distorting Nature, | 93 |
| The "New Code" Indorsed, | 94 |
| ***CLINICAL BUREAU.*** | |
| Retention of Urine with Hæmaturia. W. A. Hawley, M. D., | 95 |
| Clinical Reflections. Ad. Lippe, M. D., | 95 |
| Clinical Cases. E. W. Berridge, M. D., | 99 |
| Cases from my Note-book. Julius Schmitt, M. D., | 102 |
| Tobacco Poisoning. J. W. Thomson M. D., | 106 |
| ***BOOK NOTICES.*** | |
| Bernard: The Homœopathic Treatment of Constipation. Translated by T. M. Strong, M. D. Chatterton, | 107 |
| Helmuth: Supra-pubic Lithotomy. Bœricke & Tafel, | 107 |
| ***NOTES AND NOTICES.*** | |
| Homœopathy Ahead in San Diego—The Minneapolis Homœopathic Hospital—The Alumni Association of the N. Y. Homœopathic Medical College—The Seventh Annual Meeting of the Homœopathic Ophthalmological and Otological Society—Errata, | 108 |
| ***APPENDIX.*** | |
| Dysentery, | 17–24 |
| Cough Symptoms, | 57–60 |

